# Digital mediators as key enablers of navigation toward health in knowledge landscapes

**DOI:** 10.3325/cmj.2018.59.178

**Published:** 2018-08

**Authors:** Dina Šimunić, Antun Kerner, Srećko Gajović

**Affiliations:** 1 Department for Wireless Communications, University of Zagreb Faculty of Electrical Engineering and Computing, Zagreb, Croatia; 2 Croatian Institute for Brain Research, University of Zagreb School of Medicine, Zagreb, Croatia *srecko.gajovic@hiim.hr*

Knowledge landscapes represent virtual and real-life trajectories made by an individual in the search for health (1). The knowledge necessary to restore or maintain health is searched for not only by patients, but by all people concerned with health issues. In the digital environment, the capabilities to get health-related knowledge are greatly enhanced, with a multitude of communication directions that create an extensive geography of knowledge landscapes (2). In these landscapes, knowledge is frequently hidden by the context as an important shaping force of the landscape. Communicating the health-relevant knowledge does not mean just to communicate the relevant information (although 100% accurate), but also to appreciate the context of the communication and make the users receptive to the communicated message (3).

Here, we want to emphasize another component of knowledge landscapes – digital technology. Technology is very active in shaping knowledge landscapes, and it is the base of tremendous advancements in the digital environment. Technology provides the means by which the user and system can handle the contents of knowledge landscapes. Here, we refer to the technological agents in online landscapes as digital mediators. Digital mediators are key enablers of the navigation possibilities, in particular toward health-related knowledge. As they are not only passive bystanders, they mediate the benefits of digital health, but also contribute to the possible risks.

## Humans in the center of digital connectivity

Seamless connectivity, a state in which everyone (and everything) could be connected, has been a human dream since the ancient times. We are approaching this dream by experimenting with various features of wireless communication networks. They include massive MIMOs (Multiple-Input and Multiple-Output, a method for multiplying the capacity of a radio link using multiple transmit and receive antennas to exploit the propagation of radio waves), beamforming applications (signal-processing techniques used in sensor arrays for directional signal transmission or reception), small cells (low-powered cellular radio access nodes that operate in licensed and unlicensed spectrum in a range of 10 m to a few kilometers), and millimeter waves (the band of radio frequencies in the electromagnetic spectrum from 30 to 300 GHz). All these technologies utilize full duplex communication, which allows a simultaneous communication of the connected devices in two directions (from sender to receiver and from receiver to sender) (4).

These innovative technologies are created not only by telecommunication experts, but also by experts developing new materials and constructing new devices. At this pace, we can expect that the constantly connected applications and tools will soon become an integral part of an average person’s life. Devices applied in the health sector will be able to collect data on the health status of individual patients. Therefore, new communication networks are envisioned for the seamless connection of billions of devices around the globe. Alongside the “collector” devices, there will be devices integrated in the human body that will interfere with the bodily functions. Through physical activity in the human bodies, digital technology will not only improve the quality of people’ lives but will highly connect human society.

The largescale digital connectivity of society can be achieved relatively quickly, provided the strategic and systematic organization is synchronized interdisciplinary. At least three areas of disciplines have to be involved: technology (including novel materials, machines, telecommunications, and software experts), medicine (all kinds of medical experts), and humanities and social sciences (eg, sociologists, philosophers, and historians). Technology should establish a solid basis for exchanging the expected tremendous data amount, medicine should innovatively apply the novel diagnostic and therapeutic methods, and humanities and social sciences should provide knowledge and work on the societal aspects of the new applications.

## Tetrad of the interactive players, Humans-Artificial Intelligence-Integrated Automation-Automata (**H-AI-IA-A**)

An interactive exchange between humans and new technologies is expected, after which human activities would be complemented by digital activities of the technological entities. We want to emphasize the active role technology would have in the emerging digital environment. This is described as the tetrad of interacting players, referred to as Humans-Artificial Intelligence-Integrated Automation-Automata (H-AI-IA-A). We can expect the development of a novel anthropocentric approach, supporting humans as the center of a society that is globally-connected, not only by software, but also by human-integrated hardware. In this new setting, humans could be extended by digital communications.

The H-AI-IA-A tetrad consists of certain fixed and flexible elements. Humans are the fixed element and the center of the system. All the other elements are flexible and depend on the state-of-the-art technology. According to the Merriam-Webster dictionary (5), automaton is “a self-operating mechanism,” or “a machine or control mechanism designed to automatically follow a predetermined sequence of operations, or respond to predetermined instructions.” The automata are made in imitation of humans and therefore are located at the opposite end of the tetrad from humans, with interactive technologies in the middle.

Integrated automation would connect all kinds of automata in the human body together with the corresponding software and hardware in communication networks (which can be wireless or wired). It is foreseeable that humans will soon use integrated automation to prolong their life-span or improve their life-quality. Good examples of integrated automation in connection with automata are automaton lungs, muscles, legs, or eyes. Automaton in the heart would warn of a coming heart infarction, while automaton in the blood vessel system would report on the owner's health status and warn of any health issue. It could be connected to another automaton that administers medication directly in the body, in a quantity lower than taken by a pill (6). Together with the corresponding wireless networks and software, integrated automation would guide automata to fit exactly to the patient’s needs (which corresponds to the definition of precision medicine).

Artificial intelligence is already quite widespread – from simple calculators to flight controls and space operations. It also enables humans to improve their results and more efficiently monitor activities. Avatars based on artificial intelligence can be used to manage personal health data through an integrated digital representation of a human. In this way, they can deliver individually tailored medical interventions to a particular patient. Projects supported by the EU like eWall (7) and MyHealthAvatar (8) are very good examples of artificial intelligence’s involvement in the treatment of patients.

As avatars are a form of human impersonation, they could be considered automata as well, but virtual automata. They could be just a voice or an animated picture, but could also have a complete bodily form with features of a healthy or diseased human. An avatar with a disease can help real patients to get a better picture of their own situation and improve their disease-related narrative by associating it to that of the avatar.

## Digital mediators as decision-support aid for digital health services

McKinsey & Company’s research finds that, since 2011, venture capitalists have invested more than US $14 billion in the companies developing consumer-oriented digital and mobile health care applications in the USA (9). Ericsson has announced the launch of 5G in health care in 2020, in connection with three main predictions: that the health care will become decentralized, that the patient data will be centralized in hospitals, and that dependence on wearables and remote treatments will be increased. They report that 39% of chronic patients prefer online consultations to face-to-face meetings and that 62% agree that wearable devices will put people in charge of their own health (10).

The current state-of-the-art medical and technological knowledge has paved the way for a substitution of the “face-to-face” off-line communication between patients and physicians (micro-level) with an exchange between patients and physicians via electronic health services, not necessarily in the same country (macro-level). The digital communications alter geographical and spatial dimensions of health care and challenge traditional micro-service by interactive web-based virtual clinics. However, the question that remains still open is how to reach the patients unwilling or incapable to make the digital step? The number of internet users in 2016, according to the International Telecommunications Union statistics, was the incredible 3,385 billion, indicating that the number of people who do not trust the new technologies is getting smaller every day. The growth rate of mobile technology use in the period from 2001 to 2017 increased from less than 20% to more than 100%, while the use of fixed communications declined (11).

An example of digital health services is Health Navigator (12), which offers a package of related applications, like Health Navigator Natural Language Processing Engine. It functions like a physician or nurse listening to a patient and converts the words patients use to describe their symptoms into meaningful medical information. Another application by the same service is Health Navigator Diagnosis Engine, which generates a list of possible causes of a specific symptom or problem. This can be used by digital health assistants (eg, health bots, diagnosis symptom checkers) to interpret electronic health records. Health bots or symptom checkers are capable of making automated conversations with the patients, which can lead to better health care decisions. By using Health Navigator’s content, digital health assistants can provide an accurate, online experience close to a human conversation.

Another example of digital health service is Babylon (13), which started the operations on different continents (in UK and Rwanda) with a mission to globally offer an accessible and affordable health service. It can interpret symptoms and medical questions through a chat-bot interface and match them to the most appropriate health service. Also, there is a direct connection to a network of health care professionals able to provide medical advice.

Siemens’ Teamplay (14) is a cloud-based network aimed to bring together health care professionals in a team effort. The network connects several medical institutions and their imaging devices. Teamplay applications synchronize their activities to create the world’s biggest radiology and cardiology team. Moreover, by connecting the patient databases, the application provides tools to tackle big data. Another Siemens’ product, *syngo*.via (15), offers multi-modal reading and fast 3D results, which together with the artificial intelligence enables the experts to provide diagnosis in an open platform.

Google entered the field with its Google Suite by Google Cloud (16), which provides HIPAA (The Health Insurance Portability and Accountability Act)-compliant access to patient information and stores patient data files in a HIPAA-compliant repository on Google Drive. It keeps the data secure by using Google mobile device management and encryption, but allows authorized access to the information from remote devices (17).

## Digital mediators in knowledge landscapes

The reliability of digital mediators is important in all segments, but especially in health. Before massive health data transactions are started, the security of these transactions has to be tested, as well as the security of health data. Also, the accuracy of the personal devices delivering the body functions data has to be increased. It is of utmost importance to standardize communication protocols on the European and global level, especially related to accuracy, security, and privacy.

An emerging advancement in the digital networks is the Internet of Things (IoT). IoT includes all health-related “things” and can contribute to the current risk spectrum and increase the complexity of the health-privacy issues (18). The number of IoT connected devices, which was cca 1.6 billion in 2014, is forecasted to rise by 2020 to between 20 and 46 billion, and the number of cellular IoT devices is expected to rise to between 1.6 billion and 4.6 billion (18).

New technologies and applications change digital environment and, consequently, knowledge landscapes. Currently, very active in the social networks are internet bots, ie, software applications with automated tasks (scripts) actively involved in discussions among human participants. They could also be connected to artificial intelligence and be used to improve health care by integrating and analyzing large data amounts. This combination could contribute to making more accurate diagnoses, and thus providing quicker and better-targeted treatments.

Although knowledge landscapes are representations of humans navigating toward health, humans are not the exclusive “inhabitants” of the landscapes. There are also digital mediators, “crawling” through the landscapes and interacting with the content and human users. We have described them in the form of H-AI-IA-A tetrad, tightly connected to humans, aspiring for human qualities, and being created, controlled, and regulated by humans. Humans stay in the center of the digital society, not because they are superior, but because everything created serves human purpose.

Previously, in the off-line setting, the individual quest for health was pursued mostly through the face-to-face communication with experts or trustworthy acquaintances (eg, family members). Books or other types of printed material, also by human authors, were available as additional resources. This situation is gradually changing and is expected to significantly change in future online settings (19). Although knowledge landscapes are still predominantly navigated by humans, new digital entities are emerging, which are able to interactively influence the human’s trajectory. Therefore, when describing knowledge landscapes, we need to be aware of the non-human interactive players active there (20). As the navigation toward health is very complex, it is desirable to have these assistants at hand to digitally mediate the search for trustworthy knowledge sources and to present the knowledge in a form understandable to the user. Instead of carrying global positioning system-like help when navigating through knowledge landscapes, digital mediators could provide on-site assistance tailored to the individual needs.

However, it is difficult to perceive digital mediators as trustworthy acquaintances with the intimacy of a family member. Although digital mediators could be designed with a positive purpose, they could also play an undesirable role in social conditioning (21). The final purpose of digital mediators could be socially acceptable (ie, as a help to quit smoking), but the same style of digital mediation can lead in various directions. At the moment, there is no regulation governing digital mediation. Developing these regulations might be difficult, as the independence of the artificial intelligence from the initial creator can be a confusing factor.

In conclusion, the rapidly developing knowledge landscapes are technologically enabled by digital technology, which, through seamless connectivity and the emerging H-AI-IA-A tetrad, is becoming populated by non-human digital mediators. Digital mediators are key enablers of navigation toward health; still the ethics and governance of the digital mediators represents a controversial issue to be addressed in the near future. To seize the benefits and to solve the arising controversies, the current ethical systems could be amended in a new set of ethical principles – *corpus Hippocraticum novum*!
